# Does access to quality accreditation improve health? - Patient-level evidence from German cancer care

**DOI:** 10.1007/s10198-025-01833-z

**Published:** 2025-10-08

**Authors:** Tim Brand, Katharina Blankart

**Affiliations:** 1 CINCH Health Economics Research Center, University of Duisburg-Essen, Berliner Platz 6-8, Essen, 45127 Germany; 2 Departement of Health Professions, Bern University of Applied Sciences, Institute of Health Economics and Health Policy, Stadtbachstrasse 64, Bern, 3008 Switzerland

## Abstract

Despite medical advancements, the burden of cancer is increasing. Germany introduced the accreditation of local provider networks as organ cancer centers to enhance care quality. Treatment in these centers is associated with higher survival rates, prompting policymakers to advocate for further centralization. While an impact beyond treatment outcomes has been suggested, accreditation's broader effects on population health and potential spillovers across regions remain unclear. This retrospective cohort study evaluates the impact of local access to accredited cancer care on survival for eight cancer types. Using data from the German cancer registry (1999-2018), covering 5.3 million cases, and accreditation records, we identified 861,508 patients with local access to accredited care. Using nearest neighbor matching, incorporating individual and regional factors (e.g., accreditation in neighboring districts), these patients were matched with those who lacked accredited care in their vicinity. Cox proportional hazard models and G-Computation estimated hazard ratios (HR) and intention-to-treat effects for one-, three-, and five-year survival. Access to accredited centers significantly reduces mortality risk for breast, colon, and prostate cancer (HR: 0.87–0.96) and increases five-year survival probabilities for five cancer types (1.8–7.3 percentage points), with effects varying by disease severity. Access in neighboring districts improves survival rates for several cancer types, showing positive spillover effects beyond patients' home districts. These findings emphasize the role of accreditation in improving cancer care and suggest expanding such programs could enhance outcomes without imposing travel burdens on patients.

## Introduction

Accreditation in healthcare is a critical mechanism for ensuring that specialized healthcare providers adhere to established standards, thereby enhancing transparency and the overall quality of care [1]. In the context of oncological care, accreditation programs have been progressively implemented to address the specific challenges posed by cancer care [2, 3]. Since 2003, the German Cancer Society (GCS; German: Deutsche Krebsgesellschaft) has introduced accreditation for organ cancer centers (OCC) to define therapy guidelines, infrastructure, and documentation requirements. These programs aim to ensure the best evidence-based care and integrate services into multidisciplinary networks of inpatient and outpatient providers [3]. The accreditation programs are available to hospitals regardless of ownership type (private for-profit, private not-for-profit, or public).

Cancer incidence has doubled since 1970, positioning cancer as the second leading cause of mortality in Germany [4]. According to the World Health Organization, various forms of cancer (lung, colon and rectum) were among the top ten causes of death in high-income countries [5]. The increasing burden on the healthcare system and society caused by cancer has prompted policymakers to prioritize the accreditation of cancer centers to improve oncological care structures [6]. With the Hospital Transparency Act coming into force in 2024 [7], OCC accreditation is listed in the newly created Federal Hospital Atlas (Bundes-Klinik-Atlas), further increasing awareness for accredited care. While freedom of provider choice allows all patients in Germany to choose accredited providers, accreditation decisions of hospitals are depending on the related costs and expected benefits. When resources are scarce or the expected benefit is low, hospitals will refrain from accreditation programs, even though requirements may be achievable [8]. The decisions of hospitals about whether to seek accreditation lead to uneven access to accredited care across regions. The percentage of patients who received therapy in accredited centers in 2018 varied greatly between 29.6% for pancreatic cancer and 76.7% for breast cancer [9].

Several studies have evaluated the German accreditation program in different patient populations by cancer type, observation period, and location using survival analyses [10–14]. The evidence for the effectiveness of OCC accreditation suggests higher overall survival and longer survival time of cancer patients treated in accredited centers compared to the standard of care. Results in the international context remain inconclusive and depend on the type of accreditation program and outcome measure [15–17]. Studies that exclusively use survival analyses are based on the assumption that the ratio of mortality rates between the treatment and control group remains constant across the observation period. Accordingly, changing benefits of an intervention over time are unobservable. In addition, previous studies provide limited information about the impact of accreditation programs on quality at the regional level or a sensible distribution of accredited centers. A longitudinal analysis of hospitals found no significant differences in therapy outcomes before and after accreditation [13]. This suggests that there is no causal relationship between accreditation and quality at the organizational level, possibly due to selection effects, where hospitals demonstrating higher performance are more likely to pursue accreditation. The effects of gaining access to an accredited cancer center on patient outcomes are unknown.

The availability of accreditation might affect the demand and supply of cancer care in regional healthcare markets, all of which points to improved survival rates for patients who have accredited cancer care nearby. On the demand side, the accreditation might affect provider choice of patients. Perceived quality of services, distance from the hospital and waiting times are the main determinants of patient decision in choosing a hospital [18, 19]. Patients are willing to travel longer distances to receive care in hospitals with special therapy options, better reputation and superior quality [20, 21]. A patient will bypass the closest hospital, if the perceived difference in quality offsets the cost of travel. Accreditation status could therefore lead to a higher proportion of patients treated in hospitals that offer a better quality of care. Since cancer therapy is unlikely to occur in an emergency setting, patients can choose their provider due to their personal preferences within or outside of their district of residence. In the framework of Francetic et al. [22], this might lead to between-unit spillover effects of accreditation on cancer survival observed in nearby districts.

On the supply side, accreditation status might cause knowledge spillovers between healthcare providers. In the US, a study of 51 randomly selected referral regions demonstrates that adoption of new cancer therapies is associated with use of the same therapy by peers in the region [23]. Agha and Molitor [24] show that innovation activity in a region impacts the prescription behavior of other practitioners in the same region. Patients in regions where clinicians were lead investigator of a clinical trial were initially 36% more likely to receive the researched cancer drug. In addition to intended knowledge spillovers between network partners, the accreditation of OCCs might cause diagonal spillover effects on therapy patterns of non-accredited providers towards practicing according to accreditation standards [22].

Beyond demand and supply, regional determinants, such as professional capabilities and health care infrastructure, affect patient outcome regardless of accreditation status and should therefore be considered when assessing the impact of accreditation. Most importantly, innovation activity in cancer care is a key determinant of patient outcome. The level of innovation in cancer care is high compared to other indications, especially in the development of targeted therapies for the treatment of cancers of different tumor entities [25, 26]. Like uptake of accreditation, innovation activity is distributed unequally across regions, in part because of the high demands of specialization [27]. The introduction of new diagnostic and therapeutic procedures has improved the quality of care for most cancer patients in recent decades [28, 29]. At the local level and influenced by the available resources that determine access to innovation, patient populations have benefitted unevenly from technological progress, thereby increasing regional disparities in health outcomes [30].

In this study, we evaluate the effects of gaining access to accredited cancer care on survival probabilities at patient level for eight cancer types. We define treatment as the availability of at least one accredited cancer center at the time of diagnosis in a cancer patient’s district of residence and compare this to patients in districts without accredited cancer care. Recognizing that not all patients will seek care at an accredited cancer center once it becomes available, our approach aligns with an intention-to-treat analysis [31]. We use matching to create quasi-randomized samples of treated and untreated patients accounting for individual and regional characteristics. To account for the impact of access to accredited centers outside of patients’ district of residence, we measure accreditation in neighboring districts. We employ Cox proportional hazard models by cancer type to calculate hazard ratios of gaining access. Using G-Computation, we estimate the average treatment effect on one, three and five-year survival. Finally, we analyze the effect of available accredited centers across district borders for patients that lack accredited centers in their residential district.

We contribute to the evaluation of health care quality accreditation programs by (a) assessing the effect of access to accredited cancer care on survival rates of local patient populations, (b) accounting for regional determinants as confounders of patient outcome, and (c) considering spillovers of accredited providers in neighboring districts. The results provide a broader view on the benefit of accredited cancer care at the regional level, considering impacts of accredited cancer centers on other healthcare providers in the same region and patient mobility. Our study informs discussions on centralization of cancer care in accredited centers, currently based on accessibility predictions, neglecting local effects of accreditation on patient populations [32]. As quality assurance activities are increasing, our results can guide the development of strategies by provider organizations and health policy.

## Methods

### Institutional setting

We study access to accredited cancer care as part of the German statutory health insurance (SHI) system covering 88% of the population alongside private health insurance (PHI) for 10%, ensuring near-universal coverage with mandatory insurance participation [33]. Healthcare financing relies primarily on wage-related contributions to SHI funds, supplemented by tax subsidies, with patients having free choice of providers and no formal gatekeeping system. Cancer care is delivered through a well-developed infrastructure including population-based screening programs for breast cancer, cervical cancer, and colorectal cancer [34], alongside the accredited organ cancer centers that form the focus of this study. A disease management program has been introduced for breast cancer in 2003, defining general medical requirements in the provision of breast cancer care [35]. Healthcare delivery is characterized by high service provision levels, with one of the highest numbers of hospital beds per capita in the EU (813 per 100,000 population) and abundant physician supply, ensuring generally good geographic access to care across districts.

We focus our analysis at the district level. Germany is administratively divided into 401 districts (Kreise) defined according to the Nomenclature of Territorial Units for Statistics (NUTS), level 3, which vary considerably in size, with populations ranging from approximately 34,000 to over 1 million inhabitants.

### Data collection and sampling

We collected and combined comprehensive data on accreditation status, cancer survival, and innovation activity from five sources at the patient level by cancer type, district of residence and year of diagnosis.

To capture patient access to accredited cancer centers, we obtained anonymized data of all accredited OCCs for eight cancer types (breast, colorectal, gynaecological, head and neck, lung, neurological, pancreatic, and prostate cancer) from the GCS. OCCs are accredited networks of inpatient and outpatient healthcare providers that specialize in the diagnosis and treatment of cancer based on the latest clinical evidence and quality standards. Accreditation is initially granted by an independent auditing body, OnkoZert, and maintained through annual surveillance audits. Site-specific accreditation guidelines are established by relevant professional associations and outline standards for treatment, staffing, infrastructure, patient volume, and research requirements [3]. While treatment decisions remain the responsibility of the attending physicians, accreditation establishes overarching quality standards, such as the implementation of interdisciplinary tumor boards. GCS data includes information on district and year of accreditation spanning from 2003 to 2018. It also documents instances of loss and re-accreditation for each center, enabling the determination of the number of accredited centers by district, year, and cancer type.

To measure health outcome in oncological care, we collected patient data, sourced from the German cancer registries provided by the Robert Koch Institute [36]. We obtained 5,318,911 cases diagnosed with cancers covered by the eight cancer accreditation programs between January 1999 and December 2018. The dataset contains information such as district of residence, age at diagnosis, gender, date of diagnosis, diagnosis code according to the International Classification of Diseases, Tenth Revision (ICD-10), UICC TNM staging, and, if applicable, date of death. The four-stage UICC TNM staging system was defined by the Union for International Cancer Control (UICC) [37]. It distinguishes levels of tumor size and extent (T), lymph node involvement (N) and metastases (M). Patients with UICC TNM stage IV have the highest severity. At stage IV, the therapy goal is usually no longer a cure but a short-term prolongation of life [14]. Since 1999, the cancer registries of the federal states have reported cancer patients, though the reporting initiation varied among states. By 2009, all federal states were represented in the dataset. In the cancer registries, all dates are available at the monthly level.

We distinguish between colon and rectal cancer. Although these patient groups are treated in the same accredited centers, survival rates differ for colon and rectal cancer patients [14]. We calculated survival time in month and five-year rolling averages for cancer incidence by district to account for regional demand for cancer care while mitigating variations due to reporting quality. From the cancer registry data, we captured tumor size and extent to account for severity of the disease in all patients. In addition, we capture disease severity using the more detailed UICC TNM staging system. Since the UICC TNM status was recorded much less frequently, we restrict its use to subgroup analyses for the severity of the illness.

We consider several regional preconditions as confounders which may influence healthcare service accessibility, demand and therapy outcome [38]. The first precondition is the household income [39]. Accordingly, we collected Gross Domestic Product per capita (GDP) data, obtained from the INKAR database provided by Germany’s Federal Institute for Research on Building, Urban Affairs, and Spatial Development by district and year between 2000 and 2017.^1^ As GDP data was missing for 2018, we imputed the average GDP from the previous year.

Another precondition is the accessibility of healthcare providers that affects patients` health outcome [40, 41]. To account for regional differences in hospital infrastructure, we count hospitals and university hospitals by district and year, using the hospital index provided by the Federal Statistical Office of Germany.^2^ This dataset includes basic information for all German hospitals, such as location, ownership status, and number of beds per hospital unit. We exclude hospitals with orthopedic and/or psychiatric departments only, and hospitals without inpatient beds, as these hospitals do not offer cancer therapy.

The third precondition we consider is regional innovation activity in the same cancer type as the accreditation program, measured by publication records, as the research and development process often extends beyond patenting in medical innovation [42]. The patents or research and development expenditures of innovative companies, which are used in other innovation-related studies, typically disregard the knowledge produced outside the scope of the product development and patenting process that often involves clinicians in daily practice [43]. This knowledge helps to generate and communicate evidence from a technology’s use and gradually contribute to the diffusion of new health care technologies. For example, randomized controlled trials indicating a significant improvement in patient survival through off-label use of cancer drugs led to an 85% increase in off-label prescriptions for these drugs [44].

To measure regional innovation activity, we processed the 2020 baseline version of the US National Library Of Medicine/PubMed database, maintained by the US National Library of Medicine (NLM).^3^ PubMed is the world’s largest biomedical database and includes 33 million records on life sciences and biomedical topics, with information such as author names, publication names, publication dates, affiliated organizations, and associated clinical trials and research grants. To identify publications corresponding to cancer types for which accreditation data is available, we mapped relevant ICD codes to the structured thesaurus terminology according to NLM’s Medical Subject Headings (MeSH) (Appendix Table 6). We extracted 242,976 records published between 2000 and 2018. Given that each article on average has 12 MeSH terms assigned, we allowed a single publication to be associated with multiple cancer types [45]. Publications were linked to geocodes of the authors’ affiliated organizations at the district level using MapAffil [46].

To ensure the validity of our results and comparability to previous studies, we defined several exclusion criteria. We excluded neurological cancer patients, as they are not classified using the UICC TNM staging, which limits adjustment by cancer severity. We excluded 234,096 cases by limiting the data to each patient’s first diagnosis. We excluded 7,624 patients younger than 18 years at time of diagnosis. 642,723 patients were excluded for whom the date of diagnosis and date of death were identical, indicating that no therapy was performed. After dropping cases with missing data, the preliminary sample contains 3,162,418 patients. 861,508 patients had access to at least one accredited cancer center of the patients` cancer type in their district of residence and are considered treated. 2,300,910 patients did not have access to any accredited cancer center for their cancer type and are considered untreated.

### Matching of treatment and controls

We employed nearest neighbor matching with Mahalanobis distance and replacement using the MatchIt package version 4.5.5 in R [47], pairing each treated patient with five untreated patients of similar demographic, disease-specific, and regional characteristics. The variability in reporting quality across years, federal states, and patient categories impedes longitudinal analyses of survival rates across districts by accreditation status. Specifically, a difference-in-difference design exploiting the temporal variation in accreditation status at district level did not show feasible as for the majority of cancer types and districts, there were not enough patient cases reported to calculate time-averaged hazard ratios as outcomes and the parallel trends assumption was not met [48].

To mitigate potential biases arising from differences between patients seeking care in accredited versus non-accredited centers, we matched patients based on cancer type, diagnosis year, sex, age group (18–59 years, 60–79 years, 80 years or older), and tumor size and extent. To account for related selection and anticipation effects by providers and district level differences in quality of cancer care, we included a set of confounding variables: GDP, incidence, number of hospitals and university hospitals, publication output, and the number of accredited cancer centers in neighboring districts. Hospitals and providers in the network may have improved their practices before accreditation and may need to prepare the necessary infrastructure such as requirements for imaging [49]. Obtaining accreditation demands extensive requirements such including organizational structures, systems and procedures. Another concern is that patients may have anticipated accreditation which is unlikely. Accreditation status is typically made public only after obtaining accreditation at facilities or websites. Besides, patients typically choose a provider for their cancer treatment only after obtaining a cancer diagnosis which is typically not anticipated. Continuous variables were stratified into quartiles, except for diagnosis year, university hospitals and centers in neighboring districts. Access to a university hospital was used as a binary variable, while the count of centers in neighboring districts was included as a continuous variable to enhance sample balance. Diagnosis year was used for exact matching, so was cancer type.

We used replacement for better matching results, allowing each untreated patients to be matched to multiple treated patients with corresponding weighting. For all subgroup analyses, we repeated the matching process to achieve balance in each sample.

Table 1 presents the summary statistics for the baseline sample allowing for duplicates in the untreated group. The sample contains 861,508 treated patients and 246,240 individual untreated patients with effective sample size after weighting of 23,300 untreated patients. Patient groups vary by cancer type between 80,166 patients with lung cancer and 2,358,942 patients with breast cancer.

**Table 1:** Patient level summary statistics of matched sample including duplicates in the untreated group

	Untreated			Treated		
Variable	***N***	**%**		***N***	**%**	
Cancer type	4,307,540			861,508		
Breast	1,988,285	46%		397,657	46%	
Colon	753,385	17%		150,677	17%	
Rectum	276,650	6%		55,330	6%	
Head and Neck	261,485	6%		52,297	6%	
Pancreas	534,205	12%		106,841	12%	
Lung	66,805	2%		13,361	2%	
Gynaecological	70,715	2%		14,143	2%	
Prostate	356,010	8%		71,202	8%	
Sex	4,307,540			861,508		
Male	1,392,393	32%		279,739	32%	
Female	2,915,147	68%		581,769	68%	
Age group	4,307,540			861,508		
18–59 years	1,281,354	30%		265,222	31%	
60–79 years	2,434,951	57%		475,257	55%	
80 years and older	591,235	14%		121,029	14%	
T-status	4,307,540			861,508		
In situ	184,816	4%		36,987	4%	
1	1,510,701	35%		302,996	35%	
2	1,194,550	28%		238,727	28%	
3	974,809	23%		193,398	22%	
4	442,664	10%		89,400	10%	
	**N**	**Mean**	**SD**	**N**	**Mean**	**SD**
GDP per capita (in thousands)	4,307,540	41	19	861,508	40	17
Incidence (5-year average)	4,307,540	190	157	861,508	390	543
Hospitals	4,307,540	7	5.7	861,508	16	21
University hospitals	4,307,540	0.4	0.52	861,508	0.48	0.65
Publications (5-year average)	4,307,540	25	44	861,508	57	101
Accredited centers	4,307,540	0	0	861,508	2.4	2.4
Accredited centers in neighb. districts	4,307,540	3.3	4.7	861,508	3.8	4.9

The data used in this analysis were compiled from various sources, including accreditation data from the German Cancer Society, cancer registry data provided by the Robert Koch Institute, bibliometric data obtained from the US National Library of Medicine, amongst others. Matching of continuous variables was predominantly performed on the quartile level

Appendix Table 7 presents summary statistics for the treatment and control groups, both in the raw data and in the matched baseline sample. The matching for the baseline model creates a good balance between treatment and control group. Standardized mean differences are generally below the recommended threshold of 0.1. Exceptions are individual categories in hospital count and incidence, and the number of accredited centers in neighboring districts, indicating some remaining imbalance in the matched sample. Variance ratios for continuous variables are close to one, indicating similar variance in treatment and control group. A visualization of the balance before and after matching is presented in Appendix Fig. 1. Specifications for subgroup analyses partly cause larger standardized mean differences for regional determinants in the respective samples.

### Empirical analyses

We consider registered cancer patients living in a district with access to an accredited cancer center as the treated group. Patients residing in districts without access to an accredited center serve as the control group. Access to an accredited center is consistently defined as having at least one accredited center located within the same district. We capture patients diagnosed with cancer for eight cancer types from the introduction year of the related accreditation program until up to 15 years after the introduction in case of breast cancer and measure the treatment effect across all available years. The matching algorithm ensured the conditional exchangeability condition. That means that after controlling for confounders, the treatment assignment of access to accredited cancer care should be independent of the potential outcomes.

The health outcome we study reflects time-to-event data such that we employed Cox proportional hazard (Cox PH) models to investigate the relationship between access to at least one accredited cancer center and survival time by cancer type [50]. A hazard function is used to estimate the risk of dying at time t ($\:h\left(t\right)$):

1 $$\:h\left(t\right)={h}_{0}\left(t\right)\times\:\mathrm{e}\mathrm{x}\mathrm{p}\left({\beta\:}_{1}centers\right)$$

$\:t$ represents the survival time. The hazard function, denoted $\:h\left(t\right)$, depends on the treatment status ($\:centers$), with $\:{\beta\:}_{1}$ quantifying the treatments impact on the hazard. $\:{h}_{0}\left(t\right)$ is the baseline hazard, representing the hazard when treatment status is equal to zero (since $\:exp\left(0\right)=1$).

This survival analysis technique, which has been used in the previous assessments of accreditation programs [10–14], allows us to assess the hazard ratio (HR) as the relation between the probabilities of treated and untreated patients to die at any given time point in the observation period:

2 $$\:HR=\frac{{h}_{treated}\left(t\right)}{{h}_{untreated}\left(t\right)}$$

For the matched patients, we utilized the custom standard error estimator recommended by Austin and Cafri [51] that uses information about both multiplicity and pairing in our matched datasets. We do not use covariates after matching.

Additionally, we estimate the intention-to-treat effect of gaining access to accredited cancer care based on logistic regression estimates:

3 $$\begin{array}{l}logit\left(P(Y_{it}=1\right)={\beta}_0+{\beta}_1{centers}_i\\+{\beta}_2{\mathrm{X}}_i+{\beta}_3({centers}_i\times X_i)+{\mathit\in}_{\mathit i\mathit t}\end{array}$$

where $\:{Y}_{it}$ is a binary indicator of survival at time $\:t$ (1, 3, or 5 years) for patient $\:i$. $\:{centers}_{i}$ is the treatment indicator (= 1 if patient $\:i$ has access to at least one accredited cancer center in their district of residence, 0 otherwise). $\:\text{}X$ is the vector of matching variables including cancer entity, diagnosis year, sex, age group, tumor size and extent (T-status), GDP per capita quartile, cancer incidence quartile, hospital count quartile, university hospital access, publication output quartile, and number of accredited centers in neighboring districts. $\:{centers}_{i}\text{}\times\:{X}_{i}$ represents the interaction terms between treatment and covariates to address remaining imbalance in the matched samples. $\:{\mathit\in}_{it}$ is the error term. The model incorporates matching weights to account for the multiplicity of patients in the control group, with robust standard errors to handle the weighting structure [52].

The estimate of interest is the marginal risk difference that we obtain through G-Computation. The logic behind G-Computation is to use the fitted logistic regression model to predict survival probabilities for each individual in the sample under both the treatment and control scenarios, and then to average these predicted probabilities [53]. Robust standard errors are calculated using the delta method. Our effect estimate expresses the average difference in the probability of survival between those who had access to accredited cancer care and those who did not. Following G-Computation methodology, we use the fitted logistic regression model to predict survival probabilities for each individual under both treatment and control scenarios, then average these predicted probabilities to obtain the marginal risk difference:

4 $$\:ITT=\frac{1}{n}{\sum\:}_{i=1}^{n}\left[P\left({Y}_{it}=1|{T}_{i}=1,{X}_{i}\right)-P\left({Y}_{it}=1|{T}_{i}=0,{X}_{i}\right)\right]$$

This approach provides the average difference in survival probability between those with and without access to accredited cancer care, expressed in percentage points. We consider the survival rates one, three and five years after diagnosis as health outcome, a standard measure in cancer care [54]. Since therapy of cancer is often not curative but prolonging the life of patients by month or years, the increase of survival rate is a relevant factor. Before matching, we excluded all patients diagnosed after December 2013 to allow for a five-year follow-up period. This reduced the preliminary sample to 2,160,924 patients. 454,254 patients had access to at least one accredited cancer center in their district of residence at the time of diagnosis while 1,706,670 patients had no access.

The datasets used during this study are available online, with exception of cancer registry data and accreditation data, which we retrieved upon request from the Robert Koch institute and the GCS, respectively. Preparation of publication data was performed with SAS, version 9.4 (SAS Institute Inc.). All further processing steps and calculations were performed with R 4.3.1 (R Foundation for Statistical Computing, Vienna, Austria).

Before initiating this study, we obtained ethical approval from an Ethics Committee. The committee raised no ethical or legal objections.

## Results

### Accreditation uptake

Table 2 presents descriptive statistics on the included accreditation programs and describes the coverage of patient populations in 2018. The proportion of patients treated in accredited centers differs by cancer type [9], and is not necessarily related to the introduction year of the respective program or the overall number of accredited centers. The average probability of surviving five years after diagnosis, derived from the baseline sample, depends highly on the type of cancer and ranges from 16% for pancreatic cancer to 83% for breast cancer.

**Table 2:** Accreditation uptake, coverage of patients and survival rates by cancer type

Cancer type/accreditation type	Year accreditation program was introduced	Accredited centers by Dec. 2018 (*N* (%))	Patients treated in accredited centers by Dec. 2018 (%)	Median survival time in months (2003–2018)	Mean probability to survive 5 years after diagnosis (2003–2018)
Breast	2003	263 (18)	76.7	-^1^	0.83
Colon/Rectum	2006	280 (19)	42.6	83/88	0.57/0.58
Head and Neck	2011	52 (4)	45.9	82	0.56
Pancreas	2010	106 (7)	29.6	13	0.16
Lung	2008	64 (4)	35.8	16	0.24
Gynaecological	2008	136 (9)	46.2	-	0.7
Prostate	2008	110 (7)	44.8	-	0.8

**SOURCE** German Cancer Society, Robert Koch Institute and Federal Statistical Office of Germany; 2003–2018 **NOTE** Median survival and mean survival probabilities are calculated from baseline sample without accounting for matching weights or multiplicity

^1^ Calculation not possible because the survival rates in the follow-up period were not below 0.5

### Effects of access to accredited oncological care

For the analyses, we employed four distinct sample specifications that varied in the definitions of treatment and control groups or patient characteristics (Appendix Table 8). Each analysis used separately matched samples. The survival analyses of the baseline sample show the relative differences between the average survival probabilities of patients with at least one accredited center available in their district compared to patients in districts without an accredited center. We find significant positive effects for 3 out of 8 cancer types (Sample 1, Table 3). For breast cancer patients, having access to at least one accredited center in their district of residence is associated with a decreased hazard of death (HR = 0.93, *p* < 0.01). Similarly, for colon cancer, this access shows a significant protective association (HR = 0.96, *p* < 0.01). Access to accredited prostate cancer centers shows the largest effect with a hazard ratio of 0.87 (*p* < 0.001). The remaining cancer types do not show significant results.

**Table 3:** Estimated effect of access to at least one accredited cancer center in district of residence in comparison to no access (overall survival) using Cox proportional hazard models

	Sample 1 (Baseline)	Sample 2 (UICC I-III)	Sample 3 (No accr. in neighbor districts)
Cancer type	HR (SE)	HR (SE)	HR (SE)
Breast	0.93** (0.03)	0.98 (0.03)	0.92* (0.03)
Colon	0.96** (0.01)	0.97˙ (0.02)	0.9*** (0.02)
Rectum	0.99 (0.02)	1.01 (0.02)	1.01 (0.03)
Head and Neck	1.04 (0.04)	1.12 (0.08)	1.02 (0.05)
Pancreas	0.97 (0.03)	0.95 (0.04)	0.93** (0.03)
Lung	0.99 (0.02)	0.91*** (0.03)	0.98 (0.02)
Gynaecological	1.07 (0.04)	0.93 (0.05)	0.95 (0.06)
Prostate	0.87*** (0.04)	0.94 (0.05)	0.81** (0.07)

**SOURCE** The data used in this analysis were compiled from various sources, including accreditation data from the German Cancer Society, cancer registry data provided by the Robert Koch Institute, bibliometric data obtained from the US National Library of Medicine, amongst others; 2003–2018 **NOTE** *** *p* < 0.001; ** *p* < 0.01; * *p* < 0.05; ˙ *p* < 0.1; The exhibit presents estimates of the Cox proportional hazard models for matched samples. Robust Standard Errors are printed in parentheses. The estimation sample includes cancer patients that were diagnosed between the introduction year for the respective accreditation program and 2018. Hazard ratio (HR) interpretation: The relative chance of dying between the treated and the untreated group. A HR below 1 indicates a protective effect of the treatment

When restricting the sample to patients with UICC TNM stages I-III who are receiving curative therapy, the survival analysis reveals significant hazard ratios of 0.97 (*p* < 0.1) for colon cancer and 0.91 (*p* < 0.001) for lung cancer (Sample 2, Table 3). For all other cancer types, the survival analysis does not indicate significantly different survival rates for patients with UICC TNM stages I-III.

The logistic regression results show the impact of access to accredited cancer care in the patients` district of residence on survival probability one, three and five years after diagnosis (Table 4). The estimates represent the difference in survival probability when at least one accredited cancer center is available in the district. The analysis of the baseline sample shows a positive effect of accreditation status for five out of eight cancer types (Sample 1, Table 4). The estimates generally increase from one-year to five-year survival probability, leading to significant effects in later years rather than early after diagnosis. Only prostate cancer center accreditation shows significant estimates for all time points. Effect sizes for five-year survival probability vary between 1.8 (*p* < 0.01) percentages points for colon cancer and 7.3 (*p* < 0.1) percentage point for head and neck tumors.

Considering patients with available UICC TNM stages I-III (Sample 2, Table 4), logistic regression shows significantly higher five-year survival probability of 4.7 (*p* < 0.05) percentage points for pancreatic cancer. Probabilities for three and five-year survival after diagnosis with lung cancer was 5.7 and 5.6 (*p* < 0.01) percentage points higher, respectively. For gynaecological cancers, five-year survival probability increased by 4.2 (*p* < 0.05) percentage points in the new sample. Notably, for head and neck tumors, we find a 2.3 (*p* < 0.1) percentage points lower one-year survival probability for patients with access to accredited care.

**Table 4:** Predicted effects of access to at least one accredited cancer center in patients` district of residence on cancer survival at one, three, and five years after diagnosis

		Sample 1 (Baseline)	Sample 2 (UICC I-III)	Sample 3 (No accr. in neighbor districts)
Cancer type	Prob(Survival)	Estimate (SE)	Estimate (SE)	Estimate (SE)
Breast	1y	−0.002 (0.002)	0.00003 (0.001)	−0.0001 (0.003)
Breast	3y	−0.003 (0.004)	0.005 (0.004)	0.007 (0.007)
Breast	5y	0.007 (0.006)	0.006 (0.005)	0.008 (0.008)
Colon	1y	0.008 (0.005)	0.003 (0.004)	0.012 (0.011)
Colon	3y	0.015* (0.006)	0.007 (0.006)	0.036** (0.014)
Colon	5y	0.018** (0.007)	0.008 (0.007)	0.039** (0.014)
Rectum	1y	−0.002 (0.005)	−0.004 (0.004)	0.005 (0.013)
Rectum	3y	0.001 (0.007)	0.011 (0.008)	0.017 (0.019)
Rectum	5y	0.006 (0.007)	0.009 (0.008)	0.004 (0.018)
Head and Neck	1y	0.020 (0.039)	−0.023˙ (0.014)	−0.006 (0.037)
Head and Neck	3y	0.057 (0.044)	−0.037 (0.041)	0.048 (0.044)
Head and Neck	5y	0.073˙ (0.041)	−0.064 (0.045)	0.048 (0.044)
Pancreas	1y	−0.009 (0.024)	0.006 (0.030)	0.023 (0.026)
Pancreas	3y	0.027 (0.022)	0.030 (0.030)	0.017 (0.022)
Pancreas	5y	0.042* (0.017)	0.047* (0.024)	0.030˙ (0.018)
Lung	1y	−0.007 (0.015)	0.013 (0.017)	−0.016 (0.017)
Lung	3y	0.023˙ (0.012)	0.057** (0.017)	0.027˙ (0.014)
Lung	5y	0.028* (0.011)	0.056** (0.016)	0.025˙ (0.013)
Gynaecological	1y	−0.001 (0.009)	0.016 (0.013)	0.001 (0.016)
Gynaecological	3y	−0.001 (0.014)	0.016 (0.016)	0.010 (0.022)
Gynaecological	5y	0.001 (0.015)	0.042* (0.021)	0.012 (0.023)
Prostate	1y	0.010˙ (0.005)	−0.001 (0.003)	0.005 (0.007)
Prostate	3y	0.026** (0.010)	0.004 (0.007)	0.031˙ (0.017)
Prostate	5y	0.030** (0.011)	0.005 (0.009)	0.036˙ (0.019)

**SOURCE** The data used in this analysis were compiled from various sources, including accreditation data from the German Cancer Society, cancer registry data provided by the Robert Koch Institute, bibliometric data obtained from the US National Library of Medicine, amongst others; 2003–2013 **NOTE** *** *p* < 0.001; ** *p* < 0.01; * *p* < 0.05; **˙** *p* < 0.1; The exhibit presents estimates of the logistic regression models for matched samples. Robust Standard Errors are printed in parentheses. The estimation sample includes cancer patients that were diagnosed between the introduction year for the respective accreditation program and 2013. Regression coefficient interpretation: The deviation (in percentage points) of survival probability between the treated and the untreated group. An estimate above 0 indicates a protective effect of the treatment

### Spillovers from accredited centers in neighboring regions

Patients in the control group may still have access to accredited care outside of their district of residence. Having an accredited cancer center in the neighboring districts may bias the effect on survival in the home district of a patient for two reasons: First, patients may choose therapy in centers of neighboring districts, which might increase the survival rates in the control group. Second, accredited centers might cause knowledge spillovers across district borders and affect other providers. To assess the influence of accredited cancer centers in neighboring districts, we re-estimate effects in a restricted subsample of 1,511,328 patients that did not have access to accredited centers in neighboring districts.

The results of the survival analysis shows similar but decreased hazard ratios in comparison to the baseline model. In the new sample, access to accredited pancreatic cancer care is related to a hazard ratio of 0.93 (*p* < 0.01) that was not significant in the baseline model (Sample 3, Table 3). With exception of colon cancer, the estimates of the logistic regression analysis only change marginally. Colon cancer patients with access to accredited care in their district of residence show a 3.6 and 3.9 (*p* < 0.01) percentage point higher probability of surviving three and five years after diagnosis, respectively. These effects are 2.1% points higher than in the baseline model (Sample 3, Table 4). Due to the reduction in sample size, the logistic regression models lose statistical power, reflected in lower significance levels.

Table 5 shows the effects of access to accredited cancer centers in neighboring districts when no accredited centers are available in the district of residence. Access to accredited centers in neighboring districts is associated with improved survival rates for most cancer types. The hazard ratios range from 0.91 (*p* < 0.001) for prostate cancer to 0.96 (*p* < 0.05) for breast and colon cancer as well as for head and neck tumors. For rectum and lung cancer, hazard ratios are not significant. The logistic regression for the survival probability one year after diagnosis suggests a positive effect of accreditation status in neighboring districts for five out of eight cancer types. Effect sizes vary from 0.6% points for prostate cancer to 3.3% points for pancreatic cancer patients. We focus on one-year survival probability in this analysis to rule out other influences from changes in the patients` district of residence, like accreditation of new cancer centers.

**Table 5:** Spillover effects of access to at least one accredited cancer center in neighboring district for patients without accredited cancer center in district of residence

	Sample 4 (Cox PH)	Sample 4 (Logistic regression, 1 year after diagnosis)
Cancer type	HR (SE)	Estimate (SE)
Breast	0.96* (0.02)	0.003˙ (0.002)
Colon	0.96** (0.01)	0.015** (0.005)
Rectum	0.998 (0.02)	0.007 (0.005)
Head and neck	0.96** (0.02)	0.00006 (0.01)
Pancreas	0.94*** (0.01)	0.033** (0.012)
Lung	1.003 (0.01)	−0.002 (0.007)
Gynaecological	0.95* (0.02)	0.011** (0.004)
Prostate	0.91*** (0.02)	0.006˙ (0.004)

**SOURCE** The data used in this analysis were compiled from various sources, including accreditation data from the German Cancer Society, cancer registry data provided by the Robert Koch Institute, bibliometric data obtained from the US National Library of Medicine, amongst others; 2003–2018 **NOTE** *** *p* < 0.001; ** *p* < 0.01; * *p* < 0.05; **˙** *p* < 0.1; The exhibit presents estimates of the Cox proportional hazard models (left) and the logistic regression models (right) for matched samples. Robust Standard Errors are printed in parentheses. The estimation sample includes cancer patients that were diagnosed between the introduction year for the respective accreditation program and 2018 or 2013, respectively. Hazard ratio (HR) interpretation: The relative chance of dying between the treated and the untreated group. A HR below 1 indicates a protective effect of the treatment; Regression coefficient interpretation: The deviation (in percentage points) of survival probability between the treated and the untreated group. An estimate above 0 indicates a protective effect of the treatment

In conclusion, access to accredited care shows an impact on survival probabilities for some, but not all cancer types. Effect sizes vary by cancer type, outcome measure, and severity of illness measured by UICC TNM staging. G-Computation suggests an increasing effect with years after diagnosis. Accreditation in neighboring districts has a positive effect on survival probabilities for most cancer types included.

## Discussion

In this study, we analyzed the effect of regional access to accredited cancer care on survival probabilities of local patient populations. We find that the availability of at least one accredited cancer center in a patient`s district of residence has a positive effect on survival probabilities for breast, colon and prostate cancer. Using G-Computation, we find positive effects of accreditation on survival with colon, head and neck, pancreatic, lung and prostate cancer, with significant differences mostly observed in later years after diagnosis.

Notably, the effect for breast cancer is only significant in the survival analysis of the baseline sample but not in the logistic regression model. The survival analyses cover the entire observation period of up to 15 years for breast cancer, compared to five years in the logistic regression model. Therefore, the results indicate a higher proportion of patients achieving long-term remission or cure due to accredited care. In contrasts, the estimates for head and neck tumors, pancreatic cancer, and lung cancer are only significant in the logistic regression model, indicating short-term survival effects of accreditation, prolonging life without significantly increasing survival probabilities in later years.

Subgroup analyses for patients with UICC TNM stages I-III reveal varying effects of access to accredited care based on cancer severity. Hazard ratios for lower severity breast and prostate cancer cases are not significant, suggesting that effects in the baseline model are driven by high-severity cases. Conversely, the analysis shows a significant positive effect for lung cancer patients in earlier stages. This is supported by logistic regression, showing significant results for lung cancer while, with the exception of pancreatic cancer and gynaecological cancers, estimates for other cancer types are not significant at the 0.1 level. Notably, access to accredited care has a negative effect on one-year survival for head and neck tumors. This could be due to poorer matching in smaller patient groups. Descriptive analyses indicate greater imbalance in smaller samples, which reduces the value of matching and the statistical power of further subgroup analyses.

Excluding patients with access to accredited centers in neighboring districts typically reveals greater differences between the treatment and control groups. Patients without local access still benefit from nearby accredited centers, increasing survival probabilities in the control group. Spillover effects are confirmed for patients lacking accredited centers in their own district, with most cancer types showing positive effects from accredited centers in neighboring districts. However, rectum and lung cancers do not exhibit significant hazard ratios. When both analyses yield significant results, hazard ratios for accreditation in patients’ home districts are generally higher than those for accreditation in neighboring districts, indicating that the impact of accredited centers diminishes with distance.

For rectum cancer, we do not observe any significant effect of accreditation on patient survival. There are several potential reasons for this. These include a possible minor effect of accreditation on patients’ provider choice or generally high quality of care for rectal cancer patients. Divergent results for rectal cancer in previous studies suggest that there are unobserved determinants of outcome for this patient group [12–14].

For breast, colon and prostate cancer, for which we find positive effects in the baseline survival model, there exist organized cancer screening programs in Germany covered by statutory health insurance. These programs aim to promote early detection, which is known to improve prognosis [55]. The uniform nationwide implementation of these screening programs suggests that their impact on patient outcomes should not differ systematically between accredited and non-accredited districts. Our matching strategy controls for regional and demographic factors that might influence screening participation, supporting the interpretation that accreditation contributes independently to improved outcomes. Overall, the identified effects of accreditation on survival probabilities in our study may result from shifts of therapy locations towards accredited, high-quality providers and knowledge spillovers between accredited and non-accredited providers. Although our study design does not distinguish between the effects of actual therapy in accredited centers and knowledge spillovers, the results offer a more comprehensive view of accreditation’s impact on patient populations.

Our results show notable deviations from results of previous studies, with consistently higher hazard ratios. These differences are plausibly as we consider access to accredited care rather than actual therapy in an accredited center as treatment. Additionally, we account for regional determinants of accreditation and patient survival like innovation activity, leading to smaller estimated treatment effects.

Interpreting the results requires consideration of several factors. The treatment effects reflect the average impact of accreditation activities within patients’ districts of residence. These activities vary across districts, influenced by cancer type and year of diagnosis. In the baseline model, treated patients had access to on average 2.4 accredited centers in their district of residence. The study design does not allow for the identification of an optimal number of accredited centers per district or the marginal effects of additional accreditation. While additional subgroup analyses might target this problem, reduced sample sizes affect the statistical power of such analyses. Moreover, research suggests dynamic treatment effects of accreditation on outcome over time after initial issuance [12]. Even though not reflected in our results, these time effects should be considered when judging the value of accreditation at the organizational and regional level.

### Policy implications

Supporting therapy of patients in accredited centers might increase the benefit of accreditation for local populations. However, centralization through strict limitation of therapy to accredited centers may cause undesired side effects. Studies show that regional differences in care infrastructure and related travel distances are associated with delayed timing in diagnosis, therapy initiation and, subsequently, reduced survival time [40, 41]. Huguet [56] suggests that patients with a low socioeconomic status are particularly affected by reduced access to specialized care. Since the benefits of accreditation vary between patient groups, it might be more effective to limit the therapy for those patients to accredited centers who are expected to receive the highest benefit, such as those with high-severity breast cancer.

While our results suggest that accredited centers in neighboring districts affect the survival probabilities of patients, a continuous diffusion of accreditation programs across districts is generally desirable to increase patient outcome at the regional level and reduce travel costs for patients. The creation of provider networks with corresponding knowledge spillovers represent further expected advantages of this expansion. Pan et al. [57] demonstrate that optimizing the location and therapy capacities of healthcare providers can enhance the efficiency of resource allocation and improve equity in access to high-quality care for local patient populations. Policymakers could promote the accreditation of hospitals with the highest potential for undersupplied communities.

Widespread local care in accredited centers is predominantly sensible for common cancers. For rarer cancers, low case numbers in individual centers may reduce quality [6]. Concentrating expertise in fewer, high-volume centers may be more effective than prioritizing local therapy. Notably, accreditation may yield benefits, such as improved coordination of care and enhanced patient satisfaction, which were not captured in our analysis. Further research is warranted to explore these potential benefits comprehensively.

### Limitations

This study is underlying limitations. One limitation is the absence of exact location data for patients or healthcare providers. Measuring providers at the district level ignores distances between patients and providers as a key determinant of accessibility. As size and therefore potential travel distance varies across districts, this lack of information might introduce bias to the results. Another limitation is the waiver of time series analyses. Accreditation status and regional characteristics are measured at the time of diagnosis. Later changes, like additional accreditation activities, are not accounted for but might affect patient outcome over time. Finally, the study might be subject to selection bias. As hospitals self-select into accreditation, it is possible that hospitals that pursued accreditation at different times after introduction of the accreditation program differ in their organizational characteristics. These unobserved characteristics might affect the survival probabilities of patients rather than the accreditation status itself.

## Conclusion

In this study, we used survival analysis and G-Computation to assess the impact of regional access to accredited cancer care on survival probabilities for eight cancer types. Our findings show variability in the adoption of accreditation across different cancers and districts. Survival analyses indicate that access to accredited care improves survival for breast, colon, and prostate cancer patients. G-Computation reveals positive effects for colon, head and neck, pancreatic, lung and prostate cancer, with significant differences mostly observed in later years after diagnosis. Analysis of spillover effects suggests that patients in districts without accredited centers still gain some survival advantage from nearby accredited centers, though less than patients with local access. These results highlight the importance of accreditation in enhancing cancer care and suggest that expanding accreditation programs could improve outcomes without imposing unnecessary travel burdens on patients.

## Figures and Tables

**Fig. 1:**
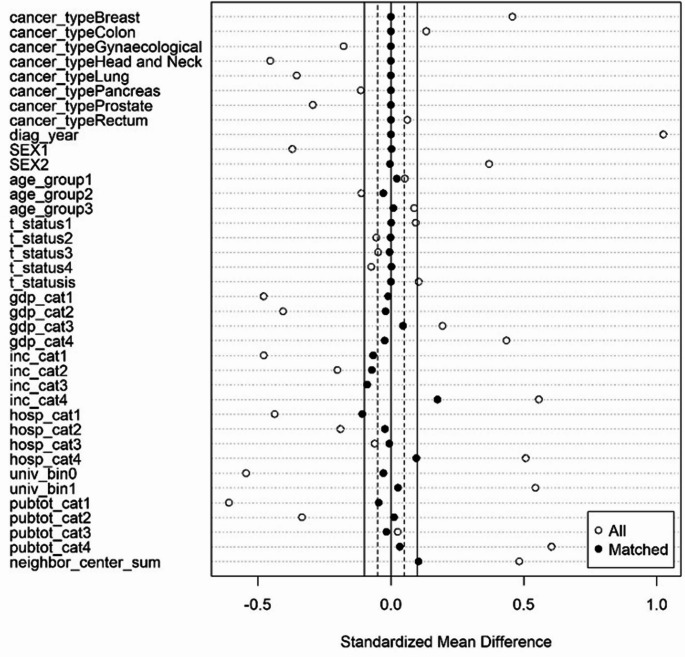
Love plot for standard mean differences in matching variables between Raw data and matched baseline sample

## Data Availability

A portion of the data has been made publicly accessible and can be found on the Open Science Framework (OSF) repository at https://osf.io/kp7zh/?view_only=d8d8b25d5f5c4018927e1bfaeefb5b3a. This includes all steps necessary to process and analyze the data, along with documentation of the corresponding publicly available datasets. The files contain author information. Due to access restrictions, some datasets used in this study are not publicly available. Access is restricted for cancer registry data and accreditation data, which we retrieved upon request from the Robert Koch institute and the German Cancer Society, respectively. Information about access to these restricted datasets may be requested from the corresponding author, subject to necessary approvals and adherence to data sharing policies. All contributors agreed to submit this paper for publication.

## Appendix

**Table 6:** Used Medical Subject Headings (MeSH) of the US national library of medicine by cancer site

Cancer site/ accreditation type	MeSH heading	Unique MeSH ID
Breast	Breast Neoplasms	D001943
Colon	Intestinal Neoplasm	D007414
Head and Neck	Head and Neck Neoplasms	D006258
Pancreas	Pancreatic Neoplasms	D010190
Lung	Lung Neoplasms	D008175
Gynaecological	Genital Neoplasms, Female	D005833
Prostate	Prostatic Neoplasms	D011471
Neurological	Nervous System Neoplasms	D009423

**Table 7:** Summary of balance between treatment and control group for raw data and matched baseline sample

		Raw data				Baseline sample			
Variable	Category	Means Treated	Means Control	Std. Mean Diff.	Var. Ratio	Means Treated	Means Control	Std. Mean Diff.	Var. Ratio
Cancer type	Breast	0.462	0.234	0.457	.	0.462	0.462	0	.
	Colon	0.175	0.124	0.134	.	0.175	0.175	0	.
	Rectum	0.083	0.065	0.062	.	0.083	0.083	0	.
	Head and Neck	0.016	0.074	-0.453	.	0.016	0.016	0	.
	Pancreas	0.016	0.029	-0.113	.	0.016	0.016	0	.
	Lung	0.061	0.145	-0.354	.	0.061	0.061	0	.
	Gynaecological	0.064	0.108	-0.177	.	0.064	0.064	0	.
	Prostate	0.124	0.221	-0.293	.	0.124	0.124	0	.
Diagnosis year		2012.928	2009.334	1.025	0.466	2012.928	2012.928	0	1
Sex	Male	0.325	0.498	-0.37	.	0.325	0.323	0.003	.
	Female	0.675	0.502	0.37	.	0.675	0.677	-0.003	.
Age group	18-59 years	0.308	0.283	0.053	.	0.308	0.297	0.023	.
	60-79 years	0.552	0.607	-0.111	.	0.552	0.565	-0.027	.
	80 years and older	0.14	0.11	0.088	.	0.14	0.137	0.009	.
T-Status	1	0.352	0.307	0.094	.	0.352	0.351	0.002	.
	2	0.277	0.301	-0.054	.	0.277	0.277	0	.
	3	0.224	0.244	-0.047	.	0.224	0.226	-0.004	.
	4	0.104	0.126	-0.073	.	0.104	0.103	0.003	.
	In situ	0.043	0.022	0.105	.	0.043	0.043	0	.
GDP per capita	1. quartile	0.13	0.291	-0.478	.	0.13	0.134	-0.01	.
	2. quartile	0.147	0.29	-0.405	.	0.147	0.153	-0.019	.
	3. quartile	0.317	0.226	0.194	.	0.317	0.295	0.046	.
	4. quartile	0.406	0.193	0.435	.	0.406	0.417	-0.022	.
Incidence	1. quartile	0.13	0.291	-0.478	.	0.13	0.153	-0.066	.
	2. quartile	0.193	0.272	-0.201	.	0.193	0.221	-0.071	.
	3. quartile	0.224	0.261	-0.088	.	0.224	0.262	-0.09	.
	4. quartile	0.452	0.175	0.556	.	0.452	0.365	0.176	.
Hospitals	1. quartile	0.14	0.291	-0.436	.	0.14	0.177	-0.107	.
	2. quartile	0.195	0.27	-0.189	.	0.195	0.204	-0.022	.
	3. quartile	0.232	0.257	-0.06	.	0.232	0.234	-0.005	.
	4. quartile	0.433	0.181	0.508	.	0.433	0.385	0.097	.
University hospitals	No	0.604	0.87	-0.544	.	0.604	0.617	-0.027	.
	Yes	0.396	0.13	0.544	.	0.396	0.383	0.027	.
Publications (5-year average)	1. quartile	0.11	0.301	-0.608	.	0.11	0.124	-0.045	.
	2. quartile	0.16	0.283	-0.334	.	0.16	0.156	0.012	.
	3. quartile	0.259	0.248	0.026	.	0.259	0.266	-0.016	.
	4. quartile	0.47	0.169	0.604	.	0.47	0.454	0.034	.
Accredited centers in neighb. Districts		3.781	1.432	0.483	2.435	3.781	3.27	0.105	1.077

**Table 8:** Specifications for treatment and control group assignment

	Sample 1 (Baseline)		Sample 2 (UICC I-III)		Sample 3 (No accr. in neighbor districts)		Sample 4 (No accr. in district of residence)	
	Treatment	Control	Treatment	Control	Treatment	Control	Treatment	Control
Accreditation in home district	yes	no	yes	no	yes	no	no	no
Accreditation in neighbor district	all	all	all	all	no	no	yes	no

Sample specifications regarding the accreditation status of home and neighboring districts between the treatment and control groups. All samples were generated through separate matching procedures, with distinct matching conducted for the Cox proportional hazards models and the logistic regression analyses

## Footnotes

1
https://www.inkar.de/, last accessed 13.06.2024.

2
https://www.statistischebibliothek.de/mir/receive/DESerie_mods_00002778, last accessed 13.06.2024.

3
https://ftp.ncbi.nlm.nih.gov/pubmed/baseline/, last accessed 13.06.2024.
